# Inhibitory and anti-adherent effects of *Piper betle* L. leaf extract against *Acanthamoeba triangularis* in co-infection with *Staphylococcus aureus* and *Pseudomonas aeruginosa*: A sustainable one-health approach

**DOI:** 10.14202/vetworld.2024.848-862

**Published:** 2024-04-19

**Authors:** Pattamaporn Kwankaew, Suthinee Sangkanu, Watcharapong Mitsuwan, Rachasak Boonhok, Udom Lao-On, Hazel L. Tabo, Tooba Mahboob, Maria de Lourdes Pereira, Jitbanjong Tangpong, Shanmuga S. Sundar, Christophe Wiart, Veeranoot Nissapatorn

**Affiliations:** 1 Department of Medical Technology, School of Allied Health Sciences, Walailak University, Nakhon Si Thammarat, Thailand; 2 School of Allied Health Sciences, Southeast Asia Water Team (SEA Water Team), World Union for Herbal Drug Discovery, and Research Excellence Center for Innovation and Health Products, Walailak University, Nakhon Si Thammarat, Thailand; 3 Akkhraratchakumari Veterinary College and Research Center of Excellence in Innovation of Essential Oil, Walailak University, Nakhon Si Thammarat, Thailand; 4 Department of Biological Sciences, College of Science and Computer Studies, De La Salle University-Dasmariñas, Cavite, Philippines; 5 Department of Pharmaceutical Biology, Faculty of Pharmaceutical Sciences, UCSI University, Kuala Lumpur, Malaysia; 6 CICECO-Aveiro Institute of Materials and Department of Medical Sciences, University of Aveiro, 3810-193 Aveiro, Portugal; 7 Department of Biotechnology, Aarupadai Veedu Institute of Technology, Vinayaka Mission’s Research Foundation Chennai Campus, Paiyanoor, Chennai, India; 8 Institute of Tropical Biology and Conservation, Universiti Malaysia Sabah, Malaysia

**Keywords:** *Piper betle* extract, *Acanthamoeba triangularis*, co-infection, keratitis, *Pseudomonas aeruginosa*, *Staphylococcus aureus*

## Abstract

**Background and Aim:**

Keratitis is a serious ocular infection often caused by pathogenic microorganisms such as *Acanthamoeba* spp. Among other harmful microbes, *Acanthamoeba* keratitis presents a particular challenge due to its resistance to conventional antimicrobial agents. *Piper betle* Linn., commonly known as betel leaf, has been traditionally used for its medicinal properties. This study aimed to assess the potential of the leaf ethanol extract of *P. betle* Linn. in the treatment of *Acanthamoeba triangularis* in monoculture and co-culture with two prevalent pathogenic bacteria, *Staphylococcus aureus* and *Pseudomonas aeruginosa*, associated with keratitis.

**Materials and Methods:**

Minimum inhibitory concentrations (MICs) of *A. triangularis*, *S. aureus*, and *P. aeruginosa* extracts in monoculture and coinfected conditions were examined. In addition, this study explored the potential of the extract in preventing *Acanthamoeba* adherence in both monoculture and co-culture environments. Scanning electron microscopy (SEM) analysis confirmed the impact of the extract on *Acanthamoeba* cell membranes, including acanthopodia. Furthermore, a time-kill kinetic assay was used to validate the amoebicidal activity of the extract against *A. triangularis* and the tested bacteria.

**Results:**

MICs for trophozoites, cysts, *P. aeruginosa*, and *S. aureus* in the monoculture were 0.25, 0.25, 0.51, and 0.128 mg/mL, respectively, whereas the MICs for *Acanthamoeba* coinfected with bacteria were higher than those in the monoculture. This extract inhibited the growth of *A. triangularis* trophozoites and cysts for up to 72 h. Moreover, *P. betle* extract effectively prevented the adherence of *Acanthamoeba* to contact lenses under monoculture conditions. SEM analysis confirmed that *P. betle* extract affects the cell membrane of *Acanthamoeba*, including Acanthopodia. In addition, the time-kill kinetic assay confirmed that the extract contained amoebicidal activity against *A. triangularis*, including the tested bacteria. Notably, *S. aureus* was more susceptible than *A. triangularis* and *P. aeruginosa* to *P. betle* extract treatment. Unexpectedly, our study revealed that *S. aureus* negatively affected *A. triangularis* in the co-culture after 3 days of incubation, whereas *P. aeruginosa* facilitated the growth of *A. triangularis* in the presence of the extract.

**Conclusion:**

This study provides compelling evidence of the anti-adhesive and anti-*Acanthamoeba* properties of *P. betle* leaf extract against *A. triangularis* under monoculture and co-culture conditions. The observed impact on *Acanthamoeba* cell membranes, coupled with the time-kill kinetic assay results, underscores the potential of *P. betle* leaf extract as a promising agent for combating *Acanthamoeba*-related infections in humans and animals.

## Introduction

*Acanthamoeba* spp. is unicellular protozoan parasites found in various natural environments, such as water, soil, and air. It has two stages: a highly durable cyst and a pathogenic trophozoite [1]. The parasite may cause infection by infiltrating the body through different channels found in humans and animals. Eye contact causes painful *Acanthamoeba* keratitis (AK). In addition, superficial skin infections can lead to cutaneous acanthamoebiasis. *Acanthamoeba* spp. can spread to the central nervous system through the bloodstream, causing granulomatous amebic encephalitis [2]. AK is a severe ocular infection that may jeopardize vision and cause vision loss. Common symptoms of AK include photophobia, ring-like stromal infiltrate, and epithelial defects [3]. *Acanthamoeba* trophozoites adhere to contact lenses using their acanthopodia, which can lead to severe vision loss and even complete blindness [4]. However, different species of the *Acanthamoeba* spp. exhibit different clinical manifestations and treatment responses [5]. *Acanthamoeba triangularis* can cause AK and may exhibit specific characteristics that make it suitable for investigating the response to AK treatment [6]. AK poses a challenge because of its high drug resistance [7]. Nonetheless, biguanides, such as polyhexamethylene biguanide and chlorhexidine, and aromatic diamidines, such as propamidine, effectively kill *Acanthamoeba* spp. [8, 9]. However, the treatment of *Acanthamoeba* infection takes approximately 2 months. In addition to addressing AK, there are other challenges. Microbial coinfections may increase the risk of adhering *Acanthamoeba* to the cornea, leading to a poor prognosis. Co-infection with *Acanthamoeba* spp. may increase the virulence of an ocular infection and cause severe symptoms in patients. *Staphylococcus aureus* and *Pseudomonas aeruginosa* commonly cause ocular infections and exhibit similar clinical presentations characterized by rapid and destructive disease. Therefore, immediate antibiotic treatment is crucial for treating these infections [10].

Medicinal plant extracts exhibit anti- *Acanthamoeba* activities and anti-virulence factors such as anti-adhesion activity against *A. triangularis* [11, 12]. *Piper betle* Linn. is a common native plant in Asia, mainly in India, China, and Vietnam. *P. betle* is a member of the family *Piperaceae* and is a dioecious, annual creeper that grows approximately 1 m in height. *P. betle* primarily grows in hotter and damper regions. It is widely used in Asia as a medicinal plant. Leaves are the most commonly used and studied part of the plant [13]. *P. betle* leaves are widely used as a chewing habit to prevent bad breath, strengthen gums, preserve teeth, cough medicine, and as an astringent. In addition, the juice of the leaves helps to treat pharyngitis, abdominal pain, and swelling. In India, *P. betle* has been used in numerous decoctions, mainly for wound healing, burns, impetigo, furunculosis and eczema, where the juice is used for pain management. Roots and fruits are associated with malaria and asthma treatment [14, 15]. *P. betle* leaves are traditionally used to treat vaginal and oral anti-candidiasis, halitosis, and conjunctivitis in Indonesia [16, 17]. In Thailand, it is widely used as a mouthwash to prevent bad breath [18]. In Malaysia, *P. betle* is considered a crucial traditional medicine for treating dental problems, headaches, and joint issues. Most traditional uses of *P. betle* are based on the antibacterial and antifungal properties of plants [14]. *P. betle* has been reported to be enriched with various potent secondary metabolites such as betel-phenol, chavicol, and other phenolic compounds [19, 20]. *P. betle* leaves have been evaluated for phytochemical analysis and have been reported to contain alkaloids, tannins, glycosides, reducing sugar, saponin, amino acids, terpenoid, and steroids [21–23]. These components have significant potential as antifungal and antibacterial agents, consistent with the traditional medicinal uses of *P. betle* [17]. However, owing to the vast recognition of its antimicrobial activities, no study has been conducted on this local plant against *A. triangularis* or co-infection of ameba with bacteria.

Therefore, the aim of this study was to investigate the anti-*Acanthamoeba* activity of *P. betle* leaf ethanol extract against *A. triangularis* and the co-infection of *A. triangularis* with *P. aeruginosa* and *S. aureus*. In addition, the anti-adhesion activity of the plant extract against these pathogens was investigated in an experimental model of contact lenses.

## Materials and Methods

### Ethical approval

This study was conducted *in vitro* and does not contain any human and/or animal participants or biological samples obtained by any of the authors in this study. All experiments were conducted according to the International Biosafety Guideline for Scientific Research at Walailak University, Nakhon Si Thammarat, Thailand (Ref. No. WU-IBC-66-020).

### Study period and location

This study was conducted from January 2022 to April 2023 at the Research Institute of Health Sciences (RIHS), Walailak University, Nakhon Si Thammarat, Thailand.

### Sample

*P. betle* plant leaf samples were collected from Phatthalung province in southern Thailand (location or Global Positioning System coordinates). We also used this same Piper betle (italic) extract that was reported in our previous study [24]. In addition, the phyto­chemical components were identified by gas chro­matography-mass spectrometry (GC-MS) analysis (Agilent Technologies 7890 B (GC) equipped with 5977A Mass Selective Detector (MS), California, USA). Hydroxychavicol, followed by phenol, 2-methoxy-4-(2-propenyl), and chavicol, were the predominant compounds detected in the leaf extract. Notably, hydroxychavicol, a key polyphenol from *P. betle* leaf, accounted for 54.61% of the total peak area observed in the *P. betle* leaf extract.

### Preparation of the *P. betle* extract

According to the protocols described by Kulnanan *et al*. [24] and Pieczykolan *et al*. [25], 200 mL of 95% ethanol was used to extract 50 g of the dry powder from *P. betel* leaf over 7 days at 25°C. The extract was filtered using Whatman filter paper (Fisher Scientific, Maidstone, UK) and evaporated under low pressure. Subsequently, the solution was air-dried at 25°C until a constant weight was achieved, and the weight was monitored daily. Finally, *P. betle* leaf extract was dissolved in 100% dimethyl sulfoxide (DMSO) and stored at 4°C until use.

### Parasites and bacterial cultures

*A. triangularis*-T4 WU 19001 was cultured in a flask containing peptone yeast extract-glucose (PYG) medium, as previously reported by Mitsuwan *et al*. [12]. The ameba was maintained at 25°C in complete darkness without moving the culture flask. Fresh culture medium was recovered from the flask every 2 days until trophozoite collection.

*A. triangularis* was cultured in PYG medium and incubated for at least 1 week without adding fresh medium to obtain 90% cysts. During this period, the parasite underwent exponential growth until reaching a maximum level of 1 x10^6^ cells/mL, following which nutrient depletion led to unfavorable conditions for trophozoite growth and encystation or cyst formation. Finally, an entirely homogenous inoculum of mature cysts was successfully harvested. Because this study focused on the interaction between *Acanthamoeba* and bacteria, PYG medium was used for culturing the bacteria. *P. aeruginosa* (ATCC10145) and *S. aureus* (ATCC25923) were cultured in PYG at 37°C for 18–24 h, washed 3 times with normal saline solution, and resuspended to a final concentration of 1 × 10^6^ colony-forming unit (CFU)/mL.

### Determination of the minimum inhibitory concentrations (MIC) of *P. betle* leaf extract

#### MIC of P. betle leaf extract against A. triangularis

The MIC of *P. betle* leaf extract against *A. triangularis* was examined using the broth dilution method. The extract was diluted in PYG medium to a final concentration of 2, 1, 0.5, 0.25, 0.125, and 0.0625 mg/mL in a microplate, and then, 100 μL of 2 × 10^5^ cells/mL trophozoites or cysts were added to each well. Chlorhexidine was used as a negative control. The extracts contained 1% DMSO. Therefore, 1% DMSO was used as a negative control. Microplates were incubated at 25°C for 24, 48, and 72 h. The parasites viability was assessed by trypan blue staining. In brief, 50 μL of the solution in each well was stained with trypan blue, and unstained (live cells) and stained (dead cells) cells were counted separately in a hemocytometer (Boeco, Hamburg, Germany). Parasite viability was calculated according to the formula percent viability = (mean live parasite/control) ×100. The minimum inhibitory concentration (MIC) value was defined as the lowest concentration that inhibited parasite viability by more than 90% compared with that in the control group.

#### MIC of P. betle leaf extract against P. aeruginosa or S. aureus

The MIC of *P. betle* extract against *P. aeruginosa* or *S. aureus* was examined using the microtiter broth dilution method. *P. betle* extract was serially diluted 2-fold in PYG medium to a final concentration ranging from 2 to 0.0625 mg/mL in a microplate, and the bacteria (100 μL, 1 × 10^6^ CFU/mL) were added into each well. Vancomycin was used as a positive control for *S. aureus*, cefotaxime, a positive control for *P. Aeruginosa*, and 1% DMSO in PYG as negative controls. The microplates were then incubated at 25°C for 18 h, and 0.03% resazurin (Thermo Fisher Scientific, Lancashire, UK) was stained to determine bacterial viability. The MIC was established as the lowest concentration that is entirely blue.

#### MIC of P. betle leaf extract against A. triangularis and S. aureus/P. aeruginosa co-infection

The MIC of *A. triangularis* when co-incubated with *P. aeruginosa* or *S. aureus* was determined using the microtiter broth dilution method. *P. betle* leaf extract was serially diluted 2-fold in PYG medium to a final concentration ranging from 2 to 0.0625 mg/mL in a microplate. *P. aeruginosa* or *S. aureus* were then added (50 μL, 4 × 10^6^ CFU/mL) to each well. *A. triangularis* was inoculated into each well (50 μL, 4 × 10^5^ cells/mL). Chlorhexidine was used as a positive control for *A. triangularis*, vancomycin was used as a positive control for *S. aureus*, cefotaxime was used as a positive control for *P. aeruginosa*, and 1% DMSO was used as a negative control. Microplates were then incubated at 25°C or 37°C for 18 h. The MIC values of *A. triangularis* and bacteria were similarly defined in Sections 2.3.1 and 2.3.2. To determine the mortality rate of *A. triangularis*, the reactions were incubated for another 3 days. Co-incubation of *P. betle leaf extract* against three microorganisms (*A. triangularis*, *S. aureus*, and *P. aeruginosa*) was performed using the same procedure.

### Impact of *P. betle* leaf extract on the adhesion of *A. triangularis* and bacteria to contact lenses under both individual and co-incubation conditions

The effects of *P. betle* leaf extract on the adhesion of *A. triangularis* to commercial soft contact lenses were performed as described by Lee *et al*. [4] with slight modifications. Briefly, 500 μL of the parasite inoculum at 2 × 10^5^ cells/mL was added to the contact lens (Duna Plus, Singapore) in 24-well plates containing 500 μL of PYG medium containing 0.5 MIC *P. betle* leaf extract of *Acanthamoeba* at the final volume and incubated at 25°C for 24 h.

The effects of *P. betle* leaf extract on reducing the adhesion of *A. triangularis*, *S. aureus*, and *P. aeruginosa* co-incubation conditions were evaluated. A parasite inoculum (4 × 10^5^ cells/mL, 250 μL) was added to 24-well plates containing 250 μL of PYG medium containing 0.5 MIC *P. betle* extract of *Acanthamoeba* in co-infection condition (0.512 mg/mL) at the final volume and incubated at 25°C for 24 h. *P. aeruginosa* (250 μL, 4 × 10^6^ CFU/mL) and *S. aureus* (250 μL, 4 × 10^6^ CFU/mL) were then inoculated on commercial soft contact lenses.

The contact lenses were washed with phosphate-buffered saline (PBS) to remove the non-adhesive cells. The lenses were vortexed after being dissolved in 500 μL of PBS in 1.5 micro centrifuge tubes (Jet Biofil, Beijing, China). Samples were counted using a trypan blue exclusion assay under an inverted microscope (Nikon, Tokyo, Japan). The study controls used 0.5 MIC chlorhexidine and ReNu fresh multipurpose solution (MPS) as positive controls and 1% DMSO as negative controls.

### Scanning electron microscopy (SEM)

SEM (Zeiss, Munich, Germany), was used to study the morphology of *A. triangularis* trophozoites following *P. betle* extract treatment under different conditions at 2.5.1 and 2.5.2. After incubation at 25°C for 24 h, PBS was used 3 times to wash the contact lens. Lenses were fixed overnight with 2.5% glutaraldehyde in PBS. Next, samples were washed with PBS. The contact lens was dehydrated in a series of ethanol solutions (20%, 40%, 60%, 80%, 90%, and 100% ethanol), mounted on aluminum stubs, and allowed to dry using a critical point dryer. Subsequently, the specimens were coated with gold and observed by SEM.

### Time-kill study

#### Time-kill kinetic study of the P. betle extract against A. triangularis

An inoculum (1 × 10^5^ cells/mL) of *A. triangularis* was grown in PYG with the *P. betle* extract at 1-, 2-, and 4-times MIC and incubated at 25°C. *A. triangularis* was grown in PYG with 1% DMSO and used as a negative control. Samples were collected after 24, 48, 72, 96, and 120 h. At each time point, 100 μL samples were serially diluted in sterile PBS. Viable counts were determined using a trypan blue exclusion assay under an inverted microscope (Nikon). The experimental results are presented as parasite mean log + standard deviation (SD). This experiment was performed in triplicate.

#### Time-kill kinetic study of P. betle extract against S. aureus and P. aeruginosa

A typical strain of *P. aeruginosa* (ATCC10145) and *S. aureus* (ATCC25923) was used to test the time-kill kinetics of *P. betle* extract. Each type of bacteria was cultivated overnight at 37°C. An inoculum (1 × 10^6^ CFU/mL) of *S. aureus* or *P. aeruginosa* was grown in PYG with the *P. betle* extract at 1-, 2-, and 4-times MIC and incubated at 37°C. Bacteria grown in PYG containing 1% DMSO were used as the negative controls. Samples were collected after 2, 4, 6, 8, 12, 18, and 24 h. At each time point, 100 μL of each sample was serially diluted 10-fold in sterile PBS. The viable cell count was calculated using the drop plate technique. Tryptic soy agar (TSA; BD Difco, NJ, USA) plates were incubated at 37°C for 18 h.

#### Time-killing kinetic study of P. betle extract against A. triangularis under S. aureus and P. aeruginosa co-incubation conditions

*A. triangularis* with *P. aeruginosa* (ATCC10145) and *S. aureus* (ATCC25923) were used to test the time-kill kinetics of *P. betle* extracts under co-incubation conditions. Each type of bacteria was cultured overnight. An inoculum (1 × 10^6^ CFU/mL) of the culture in the PYG medium was supplemented with *P. betle* extract at 1-, 2-, and 4-fold MIC and incubated at 37°C. In addition, 1% DMSO was employed as a negative control. To evaluate the growth curve of the bacteria, the suspensions were taken at 0, 2, 4, 6, 8, 12, 18, and 24 h and added to a 96-well plate (100 µL). The optical density (OD_600_) values were determined on 96-well plates using a microplate reader.

Samples were taken at 0, 2, 4, 6, 8, 12, 18, and 24 h. Each sample (100 µL) was serially diluted in sterile PBS. A drop-plate technique was used to calculate viable numbers. TSA plates were incubated at 37°C for 24 h.

### Statistical analysis

All experiments were performed in triplicate, and the data are presented as the mean SD of the measured values. All graphs, calculations, and statistical analyses were created using GraphPad Prism software, version 8.0 (GraphPad Software, San Diego, CA, USA). The data were normally distributed. The unpaired t-test was used for the mean comparison between groups, and one-way analysis of variance (ANOVA) with *post hoc* Tukey’s honest significant difference test was used for the adhesion of *A. triangularis* and bacteria to the contact lens experiment. All analyses with a p < 0.05 were considered statistically significant.

## Results

### Anti-*Acanthamoeba* and antibacterial activities of the *P. betle* extract

**Figure-1:**
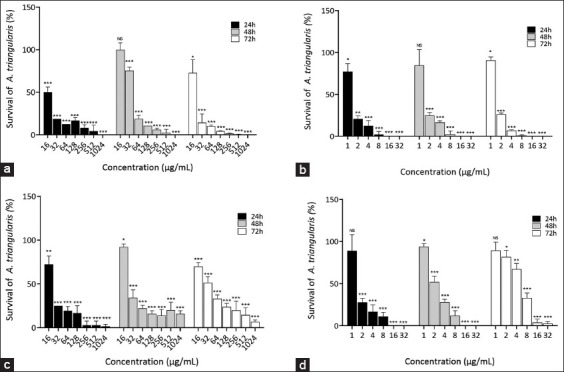
The percentage survival of *Acanthamoeba triangularis* trophozoite (a and b) and cyst (c and d) treated with *Piper betle* extract (a and c) and chlorhexidine (b and d) at different concentrations, incubated at 24, 48, and 72 h. One percent of dimethyl sulfoxide was the negative control. The experiment was performed in triplicate with three independent experiments. Error bars showed mean ± standard deviation. (* significant difference when compared between groups; *= p < 0.05, ** = p < 0.01, *** = p < 0.001).

The anti-*Acanthamoeba* activity of *P. betle* extract and chlorhexidine as a positive control against *A. triangularis* trophozoites and cysts was determined by the broth dilution method. At 24 h, the plant extract exhibited anti-*Acanthamoeba* activity against trophozoites and cysts with a similar MIC of 256 µg/mL (Table-1). However, cyst regrowth was observed due to the increase in MIC values at 48 and 72 h. Table-1 shows the MIC values of the positive controls. The percentage survival of *A. triangularis* trophozoites and cysts treated with different concentrations of *P. betle* extract at 24, 48, and 72 h was found to have a dose-dependent effect (Figure-1). The extract exhibited antibacterial activity against *S. aureus* and *P. aeruginosa*, with MIC and minimum bactericidal concentration (MBC) values shown in Table-2.

**Table-1:** Minimum inhibitory concentration (MIC) of *Piper betle* extract and chlorhexidine against *Acanthamoeba triangularis* at different time points.

Agents	MIC (µg/mL)					

	24 h		48 h		72 h	
		
	Trophozoite	Cyst	Trophozoite	Cyst	Trophozoite	Cyst
*Piper betle*	256	256	256	>1,024	256	1,024
Chlorhexidine	8	16	8	32	8	>32

**Table-2:** Antimicrobial activity of *Piper betle* extract against these selected pathogens.

Agents	Antimicrobial activity (µg/mL)					

	*A.* triangularis*		*P. aeruginosa*		*S. aureus*	

	Trophozoite	Cyst	MIC	MBC	MIC	MBC
*Piper betle*	256	256	512	>1.024	128	256
Chlorhexidine	8	16	NA	NA	NA	NA
Vancomycin	>32	>32	NA	NA	0.5	1
Cefotaxime	>32	>32	4	32	NA	NA

* Antimicrobial activity of the agents against *A. triangularis* was recorded on the MIC. NA=Not applicable, *A. triangularis*=*Acanthamoeba triangularis, P. aeruginosa*=*Pseudomonas aeruginosa, S. aureus=Staphylococcus aureus*

### Antimicrobial activity of the extract against pathogens *(A. triangularis trophozoites, P. aeruginosa, and S. aureus)* under co-infected conditions at different incubation temperatures

The MIC values of the extract against two or three microorganisms in co-incubation conditions showed that *A. triangularis* trophozoites in co-infection with *P. aeruginosa* and *S. aureus* at 25°C and 37°C ranged from 512 to 2.048 µg/mL (Table-3). Our results demonstrated that the MIC values of the extract against co-microbial incubation were higher than those of the individual pathogens. However, the MIC values measured at 25°C and 37°C, shown in 2-fold increments, did not show any significant difference.

**Table-3:** The MIC values of *Piper betle* extract against *A. triangularis* trophozoites in co-infection with *P. aeruginosa* and *S. aureus* at different incubating temperatures.

Agents	MIC values (µg/mL)					

	*A. triangularis* and *P. aeruginosa*		*A. triangularis* and *S. aureus*		*A. triangularis,* *P. aeruginosa,* and* S. aureus*	

	25°C	37°C	25°C	37°C	25°C	37°C
*Piper betle*	1.024	2.048	1.024	512	1.024	1.024
Chlorhexidine	8	8	8	8	16	16
Vancomycin	>32	>32	>32	>32	>32	>32
Cefotaxime	>32	>32	>32	>32	>32	>32

*A. triangularis*=*Acanthamoeba triangularis, P. aeruginosa*=*Pseudomonas aeruginosa*, *S. aureus*=*Staphylococcus aureus*, MIC=Minimum inhibitory concentration

**Figure-2:**
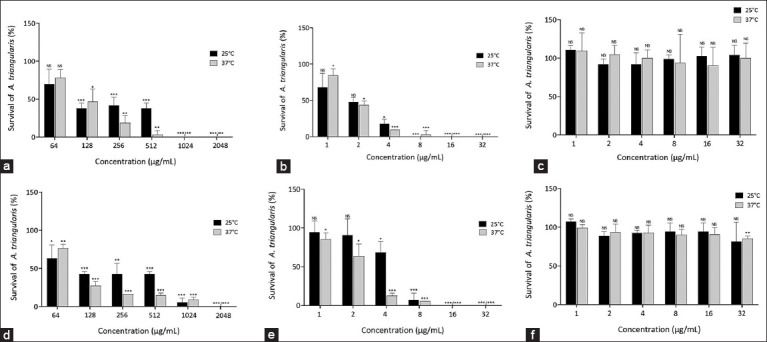
The percentage survival of *A. triangularis* trophozoite co-incubated with *S. aureus* (a-c) or *P. aeruginosa* (d-f) after treatment with *Piper betle* extract (a and d), chlorhexidine, (b and e), vancomycin (c), and cefotaxime (f). The data were presented as mean ± Standard deviation. (*, significant difference when compared between groups; *= p < 0.05), ** = p<0.01, *** = p < 0.001). *A. triangularis*=*Acanthamoeba triangularis*, *P. aeruginosa*=*Pseudomonas aeruginosa*, *S. aureus=Staphylococcus aureus*.

The percentage viability of individual *S. aureus* *or P. aeruginosa* co-incubated with trophozoites showed a dose-dependent reduction. No significant difference was observed in the percentage survival of *A. triangularis* between 25°C and 37°C under all conditions (Figures-2a and 2d). Figure-2 presents the percentage viability of the pathogen after treatment with chlorhexidine, vancomycin, and cefotaxime. Inhibition of *A. triangularis* trophozoites viability by *S. aureus* and *P. aeruginosa* co-incubated at 512 µg/mL of *P. betle* extract at 25°C (Figure-S1) was 92.31%.

**Figure-S1:**
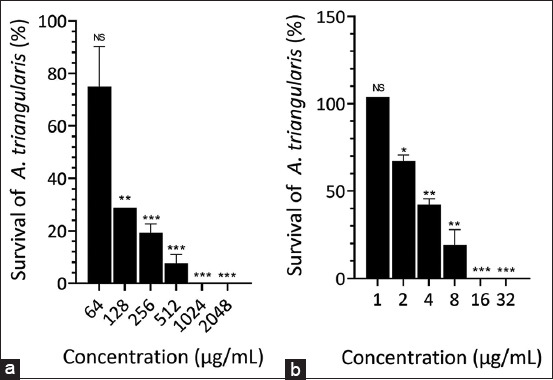
The percent survival of *A. triangularis* trophozoite co-incubate with the 2 microbes (*S. aureus* and *P. aeruginosa*) after treatment with (a) P. betle extract at 25°C and (b) chlorhexidine at 25°C. The data are presented as mean ± standard deviation. *A. triangularis*=*Acanthamoeba triangularis*, *P. aeruginosa*=*Pseudomonas aeruginosa*, *S. aureus=Staphylococcus aureus*, *P. betle=Piper betle*.

### Reduction of *A. triangularis* adhesion to the contact lens

**Figure-3:**
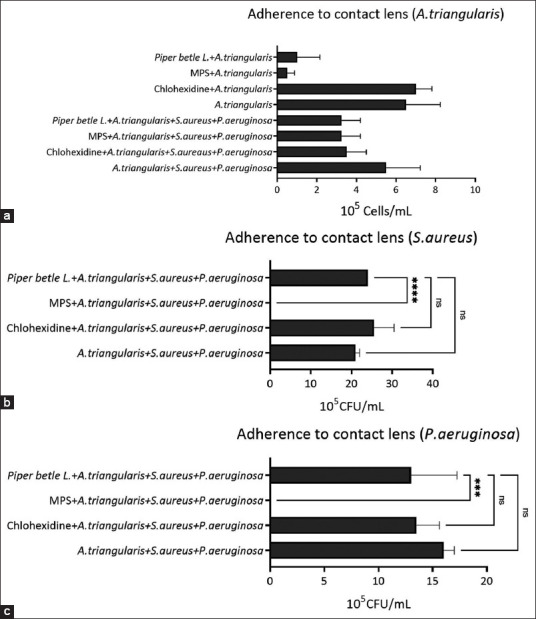
The adhesion of *A. triangularis* in monoculture and co-incubated condition after treatment with *Piper. betle* leaf extract, Chlorhexidine, and MPS (a) Effect of *P. betle L*. extract, Chlorhexidine, and MPS on adhesion of *S. aureus* in co-incubated condition at 24 h. (b) Effect of *P. betle* extract, Chlorhexidine, and MPS on adhesion of *P. aeruginosa* in co-incubated condition at 24 h. (c) The organism was treated with 0.5 MIC concentrations of the agents and incubated at 25°C for 24 h. The cells cultured in 1% DMSO in peptone yeast extract-glucose medium were used as a negative control. (Significant difference when compared between groups; *** = p < 0.001). *A. triangularis*=*Acanthamoeba triangularis*, *P. aeruginosa*=*Pseudomonas aeruginosa*, *S. aureus=Staphylococcus aureus*, MPS=Multipurpose solutions.

Figure-3a shows the effectiveness of *P. betle* extract against trophozoites adhesion on contact lens materials using an experimental model. A one-way ANOVA indicated a significant overall difference in adhesion among all lenses. Compared with the negative control, *P. betle* extract at 0.5 MIC significantly inhibited the attachment of *A. triangularis* trophozoites to the contact lenses under monoculture conditions. In addition, the positive control, MPS, showed no significant difference in reducing trophozoites adhesion compared with *P. betle* extract. *A. triangularis* trophozoites were not inhibited from adhering to contact lenses treated with 0.5 MIC chlorhexidine. Furthermore, under co-culture conditions, the number of *Acanthamoeba* trophozoites attached to contact lenses did not differ significantly across all groups, including the positive control group treated with MPS (Figure-3a).

Under co-incubation conditions, further evaluation of the efficacy of *P. betle* extract and chlorhexidine as antibacterial agents for cleaning contact lenses indicated that neither *P. betle* extract nor chlorhexidine effectively inhibited the adhesion of *S. aureus* and *P. aeruginosa* bacteria cells to the contact lenses compared with the negative control (Figures-3b and 3c).

### Effects of *P. betle* extract on *A. triangularis* by SEM

**Figure-4:**
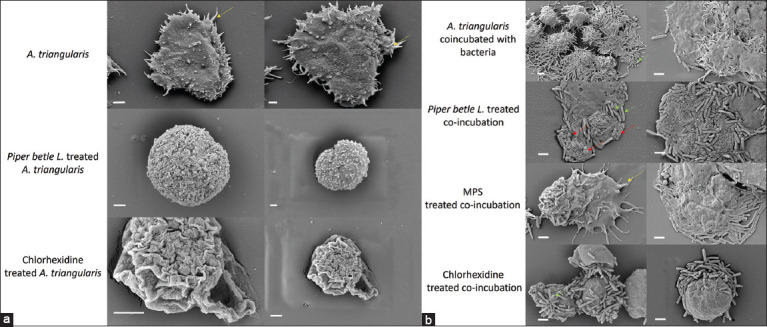
(a) Scanning electron microscope images (Magnification=5,000×–10,000×) of adherent *A. triangularis* trophozoites to contact lens showed the morphology of *A. triangularis* after being treated with *P. betle* or Chlorhexidine at 0.5 MIC. *A. triangularis* in 1% DMSO included PYG was negative control. The yellow arrow indicated acanthopodia. Bars, 1 µm. (b) Scanning electron microscope images (Magnification=5,000×−10,000×) of adherent *A. triangularis* trophozoites co-incubated with *P. aeruginosa* and *S. aureus* to contact lens showed the morphology of *A. triangularis* and bacteria. *A. triangularis* and bacteria after being treated with *P. betle* and Chlorhexidine at 0.5 MIC. *A. triangularis* co-incubated with *P. aeruginosa* and *S. aureus* in 1% DMSO included PYG was the negative control. *A. triangularis* co-incubated with *P. aeruginosa* and *S. aureus* after being treated with the contact lens multipurpose solutions was a positive control. The yellow arrow indicated Acanthopodia. The green and red arrows showed the morphology of *P. aeruginosa*. and *S. aureus*. Bars, 1 µm. *A. triangularis*=*Acanthamoeba triangularis*, *P. aeruginosa*=*Pseudomonas aeruginosa*, *S. aureus=Staphylococcus aureus*, *P. betle=Piper betle*, DMSO*=*Dimethyl sulfoxide, PYG=Peptone yeast extract-glucose.

The SEM analysis demonstrated interesting findings regarding the effects of 0.5 MIC *P. betle* extract and chlorhexidine on *A. triangularis* trophozoites and their co-incubation with *P. aeruginosa* and *S. aureus*. The trophozoites treated with *P. betle* extract showed flattened acanthopodia, leading to encystation and wrinkled cysts (Figure-4a). Co-incubation of *A. triangularis* with *P. aeruginosa* and *S. aureus* after treatment with *P. betle* extract damaged all three microorganisms (Figure-4b). Chlorhexidine treatment caused significant damage to *A. triangularis* trophozoites at 0.5 MIC. The cell membranes were visibly disrupted, with noticeable pores (Figure-4a). Co-incubation with *P. aeruginosa* and *S. aureus* further demonstrated the 0.5 MIC chlorhexidine effect, with the trophozoites assuming a round shape and a flattened, smooth surface. Bacteria appeared to cover the parasites, forming a monolayer of *P. aeruginosa* (Figure-4b). In addition, the morphology of *A. triangularis* and the co-incubated bacteria was altered by contact lens MPS. The SEM images showed a dense and shrunken mass of *Acanthamoeba* and bacteria in the treated regions (Figure-4b). The control *A. triangularis* trophozoites in PYG containing 1% DMSO showed typical morphological characteristics of acanthopodia (Figure-4a). *A. triangularis* co-incubated with *P. aeruginosa* and *S. aureus* in PYG containing 1% DMSO displayed normal shapes and surfaces of *A. triangularis* and bacteria (Figure-4b). Based on the salient normal and abnormal features in the SEM images, the results highlight the effects of *P. betle* extract, chlorhexidine, and MPS solutions on the morphological alterations of *A. triangularis* and its interactions with bacteria.

### Characterization of the *P. betle* extract mechanisms of action against *A. triangularis, P. aeruginosa*, and *S. aureus using time-kill kinetic analysis*

**Figure-5:**
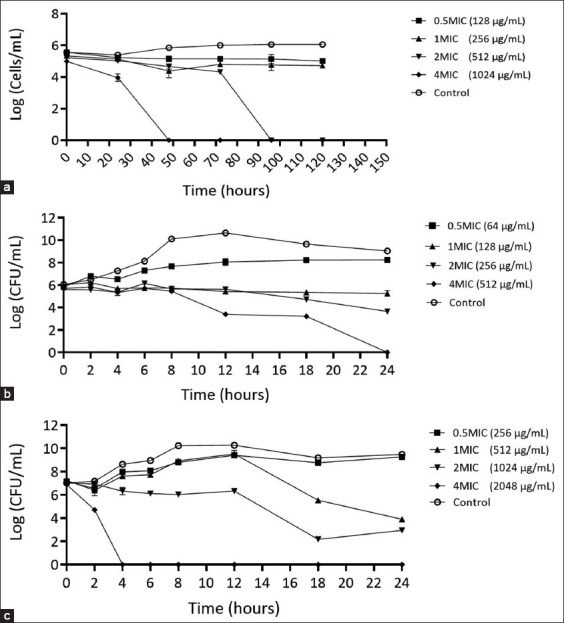
Time-kill curves illustrate the dynamic impact of mono-culture on *A. triangularis* (a), *S. aureus* (b) *P. aeruginosa* (c) after treatment with *P. betle* extract. Each microbial was treated with the extract at 4 MIC, 2 MIC, 1 MIC, and 0.5 MIC against *A. triangularis*. 1% dimethyl sulfoxide in peptone yeast extract-glucose was used as a negative control. Each symbol indicates the mean ± standard deviation. *A. triangularis*=*Acanthamoeba triangularis*, *P. aeruginosa*=*Pseudomonas aeruginosa*, *S. aureus=Staphylococcus aureus*, *P. betle=Piper betle*.

The time-kill curve analysis provided strong evidence of the antimicrobial effectiveness of *P. betle* extract against *A. triangularis*, *P. aeruginosa*, and *S. aureus* (Figure-5). The results revealed that the activity of *P. betle* extract against *A. triangularis* was dependent on the concentration, with higher concentrations leading to a greater reduction in acanthamoeba cells. At 2 MIC (0.51 mg/mL) of *P. betle* extract, *Acanthamoeba* cells were killed during the stationary phase, whereas at 4 MIC (1.02 mg/mL), *Acanthamoeba* cells were killed during the logarithmic phase (Figure-5a). For *S. aureus*, the time-kill curve revealed that the extract at 4 MIC (0.51 mg/mL) exhibited bactericidal activity against *S. aureus* at 24 h. Moreover, exposure to the extract at 2 MIC for 12 h resulted in a significant 5-log reduction in *S. aureus* viability compared with the untreated condition. At 1 MIC, the extract exhibited bacteriostatic effects on *S. aureus*, as the log CFU/mL concentration remained constant over time (Figure-5b). No colonies of *P. aeruginosa* were found at 4 MIC (2.05 mg/mL) of *P. betle* extract at 4 h. At 18 h, the extract exhibited a bactericidal effect against *P. aeruginosa*, reducing the CFU/mL starting log by more than 5 logs at 2 MIC. At 18 h, 1 MIC of the extract inhibited the growth of *P. aeruginosa* (Figure-5c).

### Characterization of the mechanisms of action of *P. betle* leaf extract against *A. triangularis* when co-incubated with bacteria using time-kill kinetic analysis

**Figure-6:**
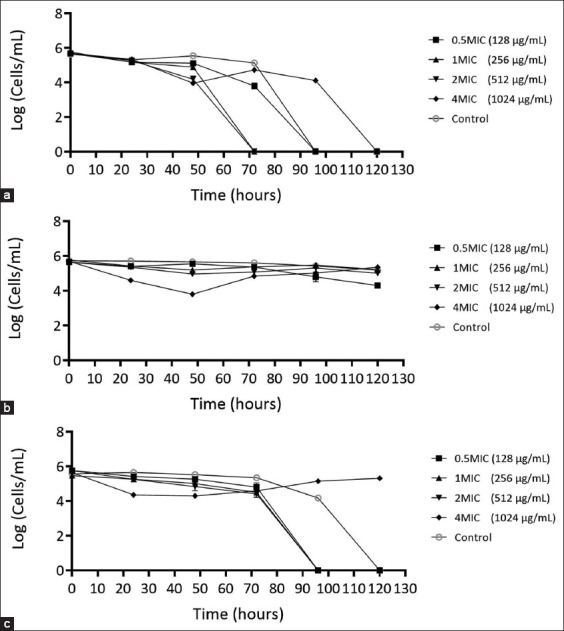
Time-kill curves of *A. triangularis* when co-incubated with *S. aureus* (a) *A. triangularis* when co-incubated with *P. aeruginosa* (b) *A. triangularis* when co-incubated with *S. aureus* and *P. aeruginosa* (c) after treat with *P. betle L*. extract. The microbial was treated with *P. betle* extract 4 MIC, 2 MIC, 1 MIC, and 0.5 MIC against *A. triangularis*. 1% dimethyl sulfoxide in peptone yeast extract-glucose was used as a negative control. Each symbol indicates the mean ± standard deviation. *A. triangularis*=*Acanthamoeba triangularis*, *P. aeruginosa*=*Pseudomonas aeruginosa*, *S. aureus=Staphylococcus aureus*, *P. betle=Piper betle*.

*P. betle* extract was administered at a concentration equivalent to the MIC of *Acanthamoeba* (1 MIC = 0.256 mg/mL) in the time-kill kinetic profile of *A. triangularis* co-incubated with *S. aureus*. After 72 h at 1 MIC and 2 MIC, the number of viable *A. triangularis* cells continuously decreased until none was observed. However, when exposed to 0.5 MIC of *P. betle* extract under co-incubation conditions, the parasite exhibited cell death at 96 h, similar to the negative control group. Surprisingly, the number of cells and mortality rate decreased slowly when the pathogens wereexposed to 4 MIC, contrary to the expectation of the fastest cell death. In addition, the decrease in the number of cells and mortality rate was the slowest compared with the negative control group. The pathogen remained undetected for up to 120 h (Figure-6a). The effect of *P. betle* extract on the growth of *A. triangularis* co-incubated with *P. aeruginosa* at different concentrations (0.5 MIC, 1 MIC, 2 MIC, and 4 MIC) was probed using time-kill kinetics. Among these concentrations (0.5 MIC (0.128 mg/mL), 1 MIC (0.256 mg/mL), and 2 MIC (0.512 mg/mL), no significant reduction was demonstrated in viable *A. triangularis* compared with that in the control group untreated with *P. betle* extract. However, at 4 MIC (1.02 mg/mL) *P. betle* extract, *A. triangularis* viability was significantly decreased by more than 2 logs at 48 h, followed by a gradual increase at 72 h. Nonetheless, the viability of *A. triangularis* was observed under all conditions. At 72 h and beyond, the parasite viability remained unchanged; therefore, no significant difference was observed between the *P. betle* extract-treated and control groups (Figure-6b). A time-kill kinetics study of *P. betle* extract was conducted to assess its efficacy against the co-incubation conditions of *A. triangularis*, *P. aeruginosa*, and *S. aureus*. The viability of *A. triangularis* treated with *P. betle* extracts at 0.5 MIC (0.128 mg/mL), 1 MIC (0.256 mg/mL), and 2 MIC (0.512 mg/mL) showed no noticeable reduction compared with the control group over 0–72 h, but expected changes came after 96 h, where viable *A. triangularis* was not observed in the co-incubated conditions with 0.5 MIC, 1 MIC, and 2 MIC test extracts. Furthermore, this observation was sustained at 120 h, when *A. triangularis* viability was not observed in the control group. On the other hand, when the three microorganisms were treated with 4 MIC (1.02 mg/mL) of *P. betle* extract, the parasite viability decreased by more than 1 log only at 24–72 h. However, after 96 h, there was an increase in the viability of *A. triangularis*, which continued until 120 h (Figure-6c).

### Characterization of the action of *P. betle* leaf extract against bacteria when co-incubated with *A. triangularis* using time-kill kinetic analysis

The time-kill curve was used to assess the efficacy of *P. betle* extract against *S. aureus* and *P. aeruginosa* in the presence of *A. triangularis*. To evaluate their antibacterial activity, the OD_600_ of the bacterial suspensions was measured. The results showed that 0.5 MIC (0.128 mg/mL), 1 MIC, and 2 MIC of *P. betle* extract against *A. triangularis* did not significantly reduce the OD_600_ of *S. aureus* compared with the control. Exposure to 4 MIC (1.02 mg/mL) of *P. betle* extract inhibited *S. aureus* growth, preventing an increase in OD_600_ over time (Figure-S2a). In contrast, at 18 h, no significant growth inhibition was observed for *P. aeruginosa* when co-incubated with *A. triangularis*, even at concentrations of 0.5 MIC, 1 MIC, 2 MIC, and 4 MIC of *P. betle* extract compared with the control (Figure-S2b). The time-kill curve was also used to evaluate the antibacterial effectiveness of *P. betle* leaf extract against *S. aureus* and *P. aeruginosa* under co-incubation conditions. The results demonstrated that 4 MIC did not reduce the OD_600_ over time, with absorbance slightly higher than that of the blank at all time points (Figure-S2c). The drop plate method confirmed the time-kill kinetics, when *S. aureus* co-incubated with *A. triangularis* and exposed to the MIC of *P. betle* against *S. aureus* co-incubated with *A. triangularis* (1.02 mg/mL) exhibited a reduction of approximately 2 logs at 4–10 h and 3 logs at 12 h compared with the control (Figure-S3a). Moreover, no colony of *S. aureus* was found at 2.04 mg/mL of *P. betle* extract in the three micro-organisms coincubated condition (Figure-S3c). *P. betle* extract (2.04 mg/mL) reduced the colony count by approximately 2 logs at 12 h compared with that in the control, with no significant difference at other time points (Figure-S3b). The viable cell counts of *P. aeruginosa* in co-incubation with *P. betle* extract at 2.04 mg/mL were not significantly different from those of the negative control (Figure-S3d).

**Figure-S2:**
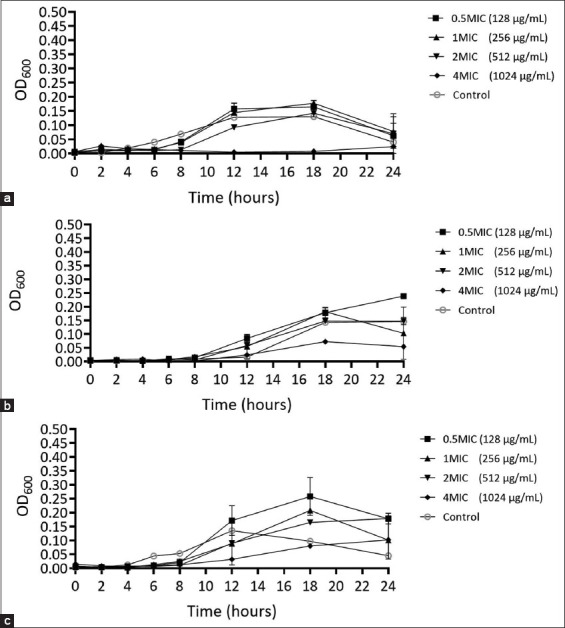
Time-kill curves of *S. aureus* when co-incubated with *A. triangularis* (a) *P. aeruginosa* when co-incubated with *A. triangularis* (b) *S. aureus* and *P. aeruginosa* when co-incubated with *A. triangularis* (c) after treated with *P. betle* extract. The microbial was treated with the extract at 4 MIC, 2 MIC, 1 MIC, and 0.5 MIC against *A. triangularis*. 1% dimethyl sulfoxide in peptone yeast extract-glucose was used as a negative control. Each symbol indicates the mean ± standard deviation. *A. triangularis*=*Acanthamoeba triangularis*, *P. aeruginosa*=*Pseudomonas aeruginosa*, *S. aureus=Staphylococcus aureus*, *P. betle=Piper betle*.

**Figure-S3:**
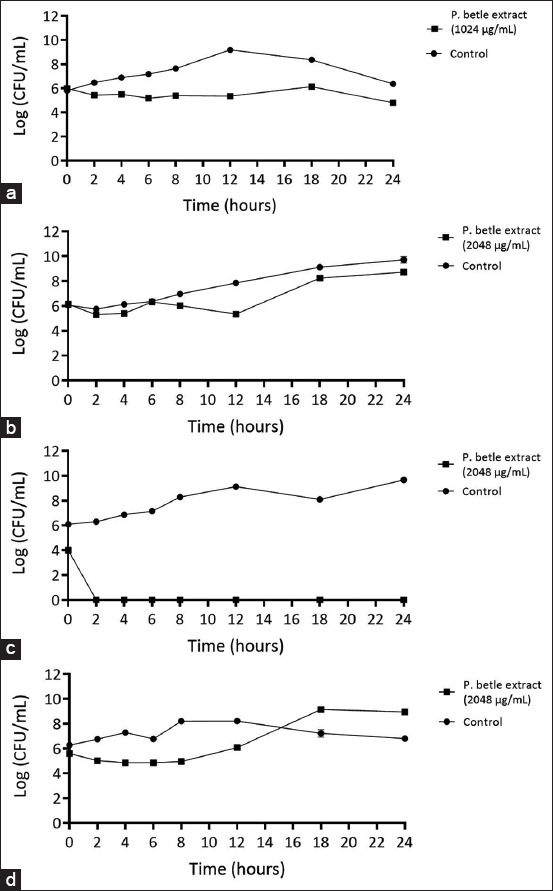
Time-kill curves of *S. aureus* when co-incubated with *A. triangularis* (a) *P. aeruginosa* when co-incubated with *A. triangularis* (b) *S. aureus* when co-incubated with *A. triangularis* and *P. aeruginosa* (c) *P. aeruginosa* when co-incubated with *A. triangularis* and *S. aureus* (d) after treated with *P. betle L*. extract. 1% dimethyl sulfoxide in peptone yeast extract-glucose was used as a negative control. The colonies were counted by the drop plate method. Each symbol indicates the mean ± standard deviation. *A. triangularis*=*Acanthamoeba triangularis*, *P. aeruginosa*=*Pseudomonas aeruginosa*, *S. aureus=Staphylococcus aureus*, *P. betle=Piper betle*.

## Discussion

Certain bacteria play a crucial role in the development of AK, and the duration of interaction between *Acanthamoeba* and bacteria can influence the severity of keratitis in humans and animals [26]. It should be noted that trophozoites feed on bacteria and reproduce by binary fission process. Previous studies by Siddiqui *et al*. [27] and El-Sayed *et al*. [28] have demonstrated the ineffectiveness of drugs in treating *Acanthamoeba*-associated infections, especially co-infection with bacteria. Selecting an appropriate medication for the treatment of AK requires the prevention and management of coinfections. Several studies have reported the antibacterial and antifungal effects of the ethanolic extract of *P. betle* leaf [15, 22, 23, 29, 30]. The aim of this study was to introduce the effectiveness of *P. betle* leaf extract to combat *A. triangularis* in both monoculture and co-culture with common pathogenic bacteria capable of causing keratitis in humans and animals, including *S. aureus* and *P. aeruginosa*. We found that *P. betle* extract contained amoebicidal activity against both *A. triangularis* trophozoites and cysts, whereas some current anti-*Acanthamoeba* drugs have limited efficacy against the cyst stage [31]. As expected, the MIC of the cysts was higher than that of the trophozoites due to their double-cell wall structure, which makes them more resistant to drug exposure [32]. Chlorhexidine, a standard anti-*Acanthamoeba* drug, was included as a positive control in this study. Over 72 h, the extract exhibited stability in the inhibition of *A. triangularis* growth for a certain period, suggesting its suitability as a potential drug for use in eye drop formulations. The *P. betle* extract inhibited *S. aureus* and *P. aeruginosa* in addition to its inhibitory activity against amoeba. Among the tested microorganisms, *S. aureus* was the most susceptible to the extract. Subsequently, a co-culture experiment involving parasites and bacteria (with the optimal bacterial growth temperature of 37°C) was conducted at both 25°C and 37°C. The *P. betle* extract inhibited both *A. triangularis* and bacteria under co-culture conditions, with no significant difference between these temperatures. Co-culturing increased the difficulty of eliminating *Acanthamoeba* when using *P. betle* or other disinfectants compared with monoculture.

*P. betle* contains several bioactive compounds such as tannins, flavonoids, and essential oils, and its pharmacological properties have been extensively studied [14, 33]. Its leaves contain essential oils with a composition typically ranging from 0.15% to 0.2% [34]. Essential oils, such as chavicol, chavibetol, carvacrol, eugenol, allylpyrocatechol, and hydroxychavicol, are believed to have inhibitory effects on various microorganisms [35]. Previous studies by Yadav *et al*. [36] and Jesonbabu *et al*. [37] on eugenol and hydroxychavicol identified their MICs against *S. aureus* in the range of 100–400 mg/L and 200 mg/L, respectively. GC-MS analysis of the ethanol extract of *P. betle* leaf used in this study revealed that hydroxychavicol was the predominant compound of the extract. In a previous study by subashkumar *et al*. [35], the extract could induce abnormal morphology in *E. coli*. The mechanism of action of hydroxychavicol has been investigated, and its ability to disrupt the microbial cell membrane has been demonstrated [30]. Eugenol can also alter cell membrane permeability, leading to intracellular leakage [38]. In the present study, SEM micrographs revealed *P. betle* leaf extract treatment-induced morphological changes in *Acanthamoeba* trophozoites from irregular to round shapes; thus, acanthopodia was shortened. However, the mechanism of cell death has not yet been characterized. Interestingly, in the co-culture of *Acanthamoeba* with *S. aureus* and *P. aeruginosa*, the ultrastructure of the ameba was different from that of the monoculture under *P. betle* leaf extract treatment. The ameba trophozoites remained in an irregular shape because of their undisrupted acanthopodia, flattened onto the contact lens, and coated with bacteria. Biofilms produced by microorganisms have been shown to help protect ameba [39]. The biofilms produced by *S. aureus* and *P. aeruginosa* may protect *Acanthamoeba* from the cytotoxic effect of *P. betle* leaf extract, which requires further investigation. In conclusion, hydroxychavicol and eugenol may be the key components in *P. betle* leaf extract that promotes anti-*Acanthamoeba* activity. It is recommended that a more comprehensive study should be conducted before any conclusions can be drawn.

Anti-adhesive effects refer to the ability of substances or treatments to inhibit the attachment of microorganisms, such as bacteria or parasites, to surfaces within the body or on medical devices, thus disrupting the initial step of infection or colonization and potentially decreasing the likelihood of biofilm formation and improving treatment outcomes [40]. In this study, *P. betle* extract at 0.5 MIC, which did not kill *Acanthamoeba*, significantly inhibited the attachment of *A. triangularis* trophozoites to contact lenses compared with the negative control. Therefore, this extract demonstrated the anti-adhesion activity of *Acanthamoeba* trophozoites to contact lenses, particularly in monoculture conditions. This finding was supported by the SEM images, which demonstrated that the ameba was stuck to the contact lens with no acanthopodia and was surrounded by bacteria. This suggests the deformity of the cell membrane and loss of adhesion organelles, which subsequently reduces the adhesion ability under *P. betle* extract treatment. Moreover, SEM data showed that *P. aeruginosa* was more predominant than *S. aureus* bacteria. Our data further suggest that the drug susceptibility of *S. aureus* was more sensitive to *P. betle* extract than to *P. aeruginosa*.

Time-kill kinetics assays are valuable for studying dynamic interactions between microbial strains and antimicrobial agents [41]. The ethanolic extract of *P. betle* has a strong effect against *A. triangularis*, *S. aureus*, and *P. aeruginosa* under monoculture conditions. In the time-kill assay of *A. triangularis* co-incubated with *P. aeruginosa* at 4 MIC, the extract was unable to kill *Acanthamoeba*, suggesting mutual symbiotic interactions between the bacteria and *Acanthamoeba*. In addition, *P. aeruginosa* may serve as a food source for *Acanthamoeba*, promoting its growth and providing essential nutrients for survival. *P. aeruginosa* is also known for its ability to form biofilms, the intricate communities of microorganisms encased in a protective matrix [42, 43], which can protect *A. triangularis* from the toxicity of the extract. A previous study by Mungroo *et al*. [44] demonstrated that the *Acanthamoeba*-bacteria co-operative relationship improved *Acanthamoeba* survival.

Unexpectedly, the time-kill assay revealed that when *A. triangularis* was co-incubated with *S. aureus* for more than 3 days, the survival of *Acanthamoeba* exposed to 4 MIC of *P. betle* extract was higher than that of the negative control group. Subsequent experiments confirmed the co-incubation of *A. triangularis* with both *S. aureus* and *P. aeruginosa*. The time-kill curve of *S. aureus* and *P. aeruginosa* co-incubated with *A. triangularis* showed that the extract effectively inhibited and eliminated *S. aureus* while paradoxically allowing *A. triangularis* survival. In contrast, *P. betle* extract at 4 MIC was ineffective in inhibiting the growth of *P. aeruginosa* during co-incubation with *A. triangularis*. This phenomenon may be explained by the fact that *Acanthamoeba* trophozoites have endosymbiotic bacteria such as *Mycobacteria*, *Legionella*, and *Pseudomonas* in their microbiomes [28, 45]. These endosymbionts may contribute to the nutrition, metabolism, and protection of the *Acanthamoeba* host from other invading microorganisms. However, further investigation should be conducted, especially in the case of this parasite, which may develop resistance if high concentrations of bacterial contaminants are added to disinfectant solutions. The presence of *P. aeruginosa* in this study suggests a protective role, potentially shielding *Acanthamoeba* from the lethal effects of *P. betle* extract [42]. On the other hand, *S. aureus* is known for its defensive properties because it produces various compounds, such as bacteriocins, peptides, and enzymes [46]. *Acanthamoeba* does not pose a threat to *S. aureus*, according to previous reports. For example, methicillin-resistant *S. aureus* can survive and proliferate in *Acanthamoeba castellanii* [47]. Moreover, *S. aureus* overgrows when co-cultured with the *Acanthamoeba polyphaga* strain [48]. This study shows *that S*. *aureus* harms *Acanthamoeba*, but its effectiveness depends on factors such as the type of *Acanthamoeba* strain, bacterial concentration in the plant extract, and parasite susceptibility to *S. aureus*-mediated killing, which deserves further study. *Acanthamoeba* infections, along with bacterial co-infections such as those caused by *S. aureus* and *P. aeruginosa*, can affect both human and animal health. Overall results suggest the potential benefits of *P. betle* leaf extract as a sustainable one-health approach, which emphasizes the interconnectedness of human, animal, and environmental health for the treatment of *Acanthamoeba* infection in single and co-infection with pathogenic bacteria.

## Conclusion

The antimicrobial activity of *P. betle* leaf extract against *A. triangularis* and co-infection with *S. aureus* and *P. aeruginosa* was demonstrated in this study. Adherence assays demonstrated that *P. betle* leaf extracts inhibited *A. triangularis* adhesion in individual cultures but did not inhibit *A. triangularis* adhesion under co-incubation conditions with bacteria. These findings imply that the presence of bacteria could play a significant role in the growth of *A. triangularis* and may provide information about the severity of AK. A potent *P. betle* extract inhibited biofilm formation and eradicated biofilms developed in co-culture with *A. triangularis* and pathogenic bacteria. This result suggests the potential benefits of *P. betle* leaf extract as a sustainable one-health approach for the holistic management of *Acanthamoeba* infection in single and co-infection with pathogenic bacteria.

## Data Availability

All data are included in the manuscript.

## Author’s Contributions

PK, SS, WM, UL, and VN: Conceived and designed the study. JT, WM, HLT, TM, MDLP, SSS, CW, and VN: Supervised the field sample and data collection. PK, SS, UL, RB, and WM: Performed the laboratory work. PK, SS, RB, WM, UL, SSS, HLT, CW, and VN: Analyzed and interpreted the data. PK, RB, SS, TM, SSS, and UL: Performed statistical analyses. VN, WM, JT, TM, MDLP, CW, HLT, and PK: Contributed to the reagents, materials, and analysis tool. PK, UL, HLT, WM, TM, RB, MDLP, SSS, and VN: Drafted and reviewed the manuscript. All authors have read and approved the final manuscript.
